# Decolonizing harm reduction

**DOI:** 10.1186/s12954-022-00593-w

**Published:** 2022-02-03

**Authors:** Gideon Lasco

**Affiliations:** University of the Philippines Diliman, Rm. 1317 3/F Palma Hall, Diliman, 1101 Quezon City, Philippines

**Keywords:** War on drugs, Drug policy, Decolonization, Harm reduction

## Abstract

In this essay, I show that notwithstanding the undeniable colonial origins of punitive drug policies around the world, such policies have figured in nationalist projects and populist platforms in various postcolonial states, and today they are viewed as local responses to the ‘drug problem.’ Instead, it is harm reduction and other efforts to reform drug policies that are seen as a colonial, or Western, imposition. I argue that to overcome such perceptions, there is a need to decolonize harm reduction alongside decolonizing drug policies. I conclude by offering recommendations toward this move, including involving Global South actors in leadership positions within the harm reduction movement, supporting pilot harm reduction programs in postcolonial states, and highlighting local scholarship.

## Background: harm reduction as a ‘western’ way of thinking

“There is a western-minded way of thinking,” Philippine Senator Vicente Sotto III said in a privilege speech in 2015, “that seeks to minimize the harm done to others by using the so-called Harm Reduction strategy” [1]. He had used the speech to denounce a pilot needle-syringe program in Cebu City where injecting drug use had been heavily linked to the rise of HIV cases, and was ultimately successful in doing so [2].

Declared Sotto—currently Senate President—in the same speech well over a year before Rodrigo Duterte became president and started his war on drugs: “Western solutions are not necessarily fit for our Eastern mind-set. We are losing hundreds of thousands of our youth to drugs and we adopt something as disastrous as this Harm Reduction strategy!” [1].

I am reminded of his statements amid growing calls to decolonize drug policy [3]. On one hand, it cannot be denied that drug policy today—including its most prohibitionist and punitive features—can be traced to colonial drug control regimes [3, 4]. But how might the attempt to decolonize drug policy in these terms resonate with how drug policy has been viewed vis-a-vis colonialism in postcolonial states like the Philippines? In this commentary, I argue that punitive drug policies are seen as local responses to the so-called ‘drug problem’ and it is actually efforts to reform drug policy—including harm reduction—that are seen as a colonial imposition. If so, then one way to overcome this perception is to decolonize harm reduction itself, and I will conclude by offering some recommendations toward this direction.

## Anti-colonial and nationalist framings of drug policy

Senator Sotto is not alone in framing harm reduction and drug policy reform in general as Western, or Euro-American, ideas. Rodrigo Duterte himself has routinely dismissed the moral authority of European and American officials to criticize his murderous drug war by invoking not just cultural differences between the East and the West, but the latter’s colonial atrocities, in the Philippines and elsewhere [5].

Beyond the Philippines, other regional leaders have framed opposition to and criticism of punitive draconian drug policies as foreign, particularly Western, interference, from Thaksin Shinawatra declaring that “the UN is not my father” is 2003 to Indonesian president Joko Widodo framing his hardline approach to drugs as “a show of national sovereignty and rejection of imported “Western” human rights concepts” [6, 7]. These statements reflect broader public sentiments: For instance, in Vietnam, “people do not accept harm reduction not because they think it is ineffective, but because of ideology…When drug use is constructed as a legacy of colonialism or as the bad remnants of capitalism, the fight against this problem must be seen as part of the class struggle” [8]. And in Northern Myanmar, “social mobilization around drug issues may be in tension with international harm reduction models of best practice” [9].

Conversely, hardline approaches to drugs are presented by their proponents as indigenous or national responses, and as consistent with ‘Asian values’ or ‘Asian democracy’ [10]. Indeed politicians in regions like Southeast Asia have embraced drug control as part of nationalist projects or populist performances [11]. As Lasco (2020) notes: “the nationalist framings of drugs, condemnations from foreign governments, UN agencies, and international human rights organizations can reinforce the narratives of populists and unwittingly play into the divisions they forge” [12].

It has not helped that the case studies most often invoked to support harm reduction are from Europe and North America—for instance, Portugal’s much-touted harm reduction paradigm or Netherlands’ long-standing decriminalization approach. Regardless of the merits of these programs, they are vulnerable to being easily dismissed (as I have witnessed in various public fora) as “not applicable in our local context.”

## Toward decolonizing harm reduction

All of the above tell us that simply labelling the global drug policy regime as a vestige of colonialism, and equating harm reduction as a step toward ‘decolonization,’ is not enough, especially to the Global South audiences who have long been resentful of colonialism (albeit in ways that may not align with today’s calls for decolonization). If harm reduction is to gain popular traction and political acceptance in countries like the Philippines, that we must likewise reflect on how we can decolonize and *indigenize* it, engaging with and addressing the particular ways in which colonization is viewed in those countries.

Thus, alongside the recommendations offered by Daniels et al. (2021) and their call for “decriminalization, decarceration, divestment and redirection” [3], here are some of the steps that we can take toward this direction:

First we need to acknowledge that indigenous efforts to resist colonial drug policies are as old as colonialism itself, and long precede the contemporary ‘decolonization turn.’ In Bolivia, for instance, the punitive drug regime has long been viewed as a colonial imposition, owing in part to prohibitionist policies on the coca leaf that are in stark contrast with people’s customs on and experiences with coca leaf consumption [13]. In Singapore, counter-discourses to the view of “drugs are evil” have been raised by popular outlets like the *Singapore Straits Times* since the early 1900s [11]. In the Philippines, accounts of drug use before the punitive drug regime—for instance, by national hero Jose Rizal—have been recovered by historians to interrogate the ways in which people who use drugs are stigmatized today [14]. If they are properly recognized as such, these accounts can strengthen the argument for decolonizing drug policy and decenter its provenance as coming from the ‘West’ or from international organizations.

Secondly, we need to support local harm reduction efforts, including local scholarship and programs that address local harm reduction needs. For instance, pilot programs on methamphetamine that have found success in Indonesia [15] may be far more compelling for Southeast Asian settings than invocations of programs in Portugal and elsewhere. Crucially, these efforts must be aligned with culturally-legible values; What Khuat and colleagues (2012) note of Vietnam can be said of other settings: “To promote the wider acceptance of harm reduction among police as well as among the community, first, and conceptually underpinning every other initiative, the concept of community safety must be expanded to include community health” [8]. Without such efforts, even local proponents of harm reduction programs can feel a disjuncture akin to that articulated by one of Chheng and colleagues’ (2012) informants in Cambodia: “Policies were written by an external consultant. They cannot understand 100% of the real Cambodian context. Sometimes the way that they designed the intervention does not fit with the Cambodian context” [16].

Third, and also in keeping with broader calls to decolonize global health and academia in general [17], we need to involve Global South voices in leadership roles in harm reduction and drug policy—from advisory boards of civil society organizations and funding agencies to editorial boards of academic journals. Multi-sited research that involves global south settings must invest in capacity building and treat locals as partners, not just as sources or gatherers of data. Beyond viewing regions like Southeast Asia as places where punitive or retrogressive drug policies need to be challenged, we also need to view them as sources of experience, expertise, and knowledge for advancing drug policy reforms around the world.

In sum, decolonizing drug policy *necessary involves decolonizing the ways we have sought to reform it.* Fortunately, if harm reduction were to be true to its principles of “respecting the rights of people who use drugs” and “commitment to social justice and collaborating with networks of people who use drugs” [18], then this move is already underway and need only be encouraged and reinforced.

## Data Availability

Not applicable.
